# Supplementary figures and images for: CAMDA 2023: Finding patterns in urban microbiomes
Source: Front Genet. 2024 Nov 25;15:1449461. doi: 10.3389/fgene.2024.1449461 (PMC11625776; doi:10.3389/fgene.2024.1449461)

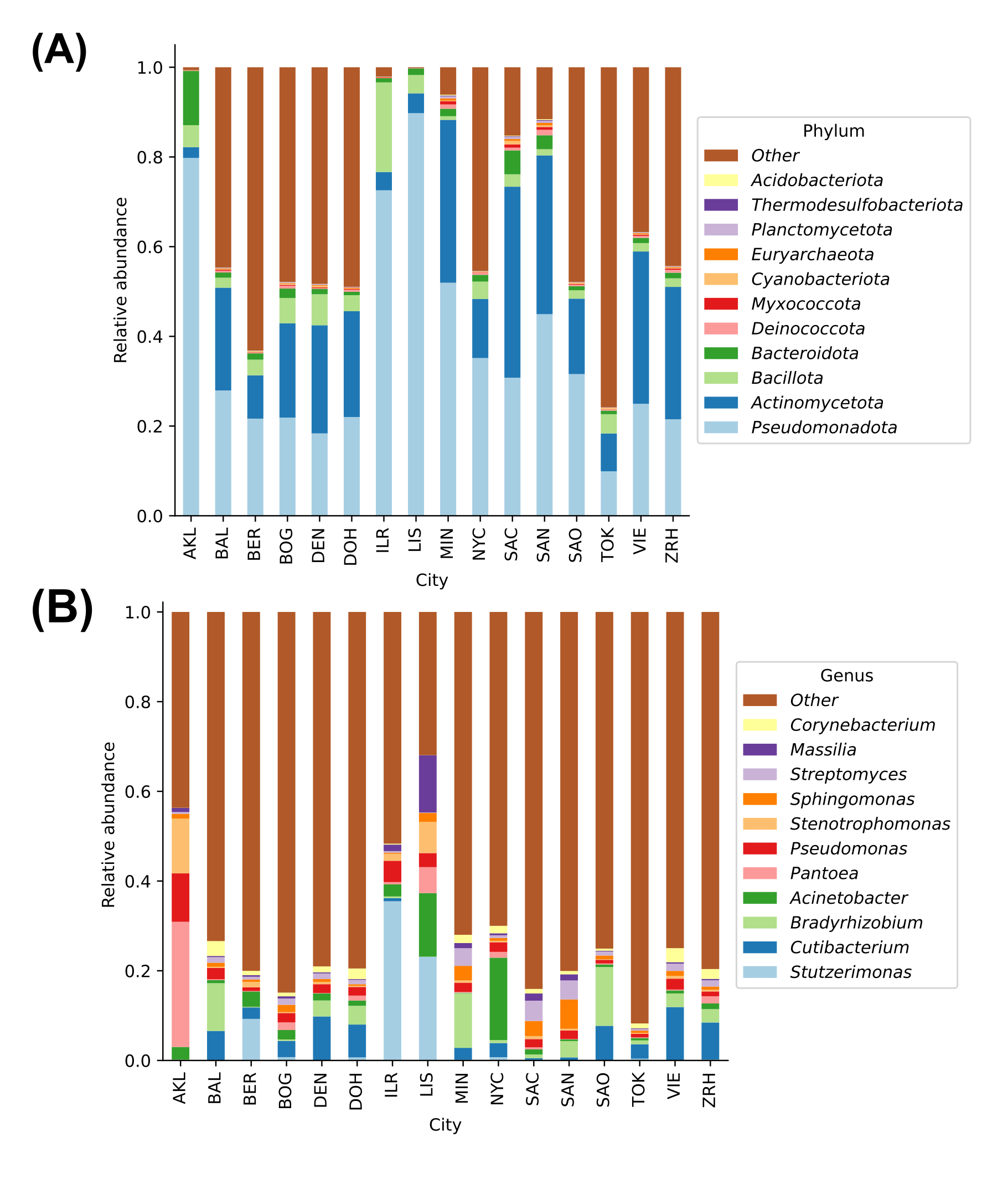

Supplement: Supplementary file 1 [file Image1.tiff]
