# Supplementary material for: CAMDA 2023: Finding patterns in urban microbiomes
Source: Front Genet. 2024 Nov 25;15:1449461. doi: 10.3389/fgene.2024.1449461 (PMC11625776; doi:10.3389/fgene.2024.1449461)
Supplement: Supplementary file 2 [file DataSheet1.pdf]

Supplementary Material

1 SUPPLEMENTARY DATA

1.1 Most differential OTUs

Table S1: We deemed an OTU to be **most differential** if it is differential for more than 10 city comparisons; i.e. if it is selected as differential for at least 11 pairs of cities. With this definition we obtained the following table, where we present the ID of the identified OTUs, the number of times it was identified as differential, and its taxonomic classification (up to Genera).

OTU	Comparisons	Kingdom	Phylum	Class	Order	Family	Genus
1280	75	Bacteria	Bacillota	Bacilli	Bacillales	Staphylococcaceae	Staphylococcus
1747	54	Bacteria	Actinomycetota	Actinomycetes	Propionibacteriales	Propionibacteriaceae	Cutibacterium
316	46	Bacteria	Pseudomonadota	Gammaproteobacteria	Pseudomonadales	Pseudomonadaceae	Stutzerimonas
288000	46	Bacteria	Pseudomonadota	Alphaproteobacteria	Hyphomicrobiales	Nitrobacteraceae	Bradyrhizobium
297	40	Bacteria	Pseudomonadota	Hydrogenophilia	Hydrogenophilales	Hydrogenophilaceae	Hydrogenophilus
1980928	33	Viruses	Uroviricota	Caudoviricetes	Herelleviridae	Sepunavirus	
2596915	27	Bacteria	Bacteroidota	Cytophagia	Cytophagales	Hymenobacteraceae	Hymenobacter
1458307	26	Bacteria	Pseudomonadota	Alphaproteobacteria	Rhodobacterales	Roseobacteraceae	Octadecabacter
1528099	25	Bacteria	Actinomycetota	Actinomycetes	Mycobacteriales	Lawsonellaceae	Lawsonella
565	24	Bacteria	Pseudomonadota	Gammaproteobacteria	Enterobacterales	Enterobacteriaceae	Atlantibacter
1982251	19	Viruses	Uroviricota	Caudoviricetes	Pahexavirus		
29370	16	Bacteria	Bacillota	Clostridia	Eubacteriales	Lachnospiraceae	Lacrimispora
225148	16	Bacteria	Chlamydiota	Chlamydiia	Parachlamydiales	Rhabdochlamydiaceae	Candidatus Rhabdochlamydia
2713640	16	Viruses	Artverviricota	Revtraviricetes	Ortervirales	Caulimoviridae	Cavemovirus
49319	15	Bacteria	Actinomycetota	Rubrobacteria	Rubrobacterales	Rubrobacteraceae	Rubrobacter
546871	15	Bacteria	Actinomycetota	Actinomycetes	Propionibacteriales	Nocardioidaceae	Friedmanniella
712435	14	Bacteria	Bacteroidota	Bacteroidia	Bacteroidales	Porphyromonadaceae	Porphyromonas
22	12	Bacteria	Pseudomonadota	Gammaproteobacteria	Alteromonadales	Shewanellaceae	Shewanella
566	12	Bacteria	Pseudomonadota	Gammaproteobacteria	Enterobacterales	Enterobacteriaceae	Pseudescherichia
38304	12	Bacteria	Actinomycetota	Actinomycetes	Mycobacteriales	Corynebacteriaceae	Corynebacterium
165179	11	Bacteria	Bacteroidota	Bacteroidia	Bacteroidales	Prevotellaceae	Prevotella
262209	11	Bacteria	Actinomycetota	Actinomycetes	Micrococcales	Intrasporangiaceae	Janibacter

2086471	11	Bacteria	Bacteroidota	Cytophagia	Cytophagales	Hymenobacteraceae	Adhaeribacter
2939494	11	Bacteria	Bacillota	Bacilli	Bacillales	Planococcaceae	Rummeliibacillus

1.2 Classification models with abundance tables

abundance	Kingdom	Phylum	Class	Order	Family	Genus	Fold presence
855575815	Bacteria	Pseudomonadota	NA	NA	NA	NA	1 1 1 1 1
618757355	Bacteria	Pseudomonadota	Gammaproteobacteria	NA	NA	NA	1 1 1 1 1
227223542	Bacteria	Pseudomonadota	Gammaproteobacteria	Pseudomonadales	Pseudomonadaceae	Stutzerimonas	1 1 1 1 1
184708059	Bacteria	Bacillota	NA	NA	NA	NA	1 1 1 1 1
157476883	Bacteria	Pseudomonadota	Alphaproteobacteria	NA	NA	NA	1 1 0 0 0
148311860	Bacteria	Bacillota	Bacilli	NA	NA	NA	1 1 1 1 1
146397481	Bacteria	Pseudomonadota	Gammaproteobacteria	Enterobacterales	NA	NA	1 1 1 1 1
132192372	Bacteria	Pseudomonadota	Gammaproteobacteria	Moraxellales	NA	NA	1 1 1 1 1
132192372	Bacteria	Pseudomonadota	Gammaproteobacteria	Moraxellales	Moraxellaceae	NA	1 1 1 1 1
126935116	Bacteria	Bacillota	Bacilli	Bacillales	NA	NA	1 1 1 1 1
117201018	Bacteria	Pseudomonadota	Gammaproteobacteria	Moraxellales	Moraxellaceae	Acinetobacter	1 1 0 0 1
100087449	Bacteria	Actinomycetota	Actinomycetes	Propionibacteriales	NA	NA	1 1 1 1 1
79516422	Bacteria	Actinomycetota	Actinomycetes	Propionibacteriales	Propionibacteriaceae	NA	1 1 1 1 1
72833080	Bacteria	Actinomycetota	Actinomycetes	Propionibacteriales	Propionibacteriaceae	Cutibacterium	1 1 1 1 1
64434208	Bacteria	Pseudomonadota	Alphaproteobacteria	Hyphomicrobiales	NA	NA	1 1 0 0 1
41773446	Bacteria	Bacillota	Bacilli	Bacillales	Bacillaceae	Lysinibacillus	1 1 1 1 1
39764946	Bacteria	Actinomycetota	Actinomycetes	Micrococcales	Micrococcaceae	NA	1 1 1 1 1
38340331	Bacteria	Bacillota	Bacilli	Bacillales	Staphylococcaceae	NA	1 1 1 1 1
37537514	Bacteria	Pseudomonadota	Gammaproteobacteria	Enterobacterales	Erwiniaceae	NA	1 1 1 1 1
37527310	Bacteria	Bacillota	Bacilli	Bacillales	Staphylococcaceae	Staphylococcus	1 1 1 1 1
36421196	Bacteria	Pseudomonadota	Alphaproteobacteria	Sphingomonadales	NA	NA	1 1 0 1 0
36046769	Bacteria	Pseudomonadota	Gammaproteobacteria	Xanthomonadales	NA	NA	1 0 1 1 0
35017217	Bacteria	Pseudomonadota	Gammaproteobacteria	Xanthomonadales	Xanthomonadaceae	NA	1 0 1 1 0
30275392	Bacteria	Pseudomonadota	Gammaproteobacteria	Enterobacterales	Erwiniaceae	Pantoea	1 1 0 1 1
29382471	Bacteria	Pseudomonadota	Alphaproteobacteria	Sphingomonadales	Sphingomonadaceae	NA	1 1 0 1 1
27966060	Bacteria	Pseudomonadota	Alphaproteobacteria	Hyphomicrobiales	Nitrobacteraceae	NA	1 1 1 1 1
26695213	Bacteria	Pseudomonadota	Alphaproteobacteria	Hyphomicrobiales	Nitrobacteraceae	Bradyrhizobium	1 1 1 1 1
24460060	Bacteria	Actinomycetota	Actinomycetes	Mycobacteriales	Corynebacteriaceae	NA	1 1 1 1 1
24460060	Bacteria	Actinomycetota	Actinomycetes	Mycobacteriales	Corynebacteriaceae	Corynebacterium	1 1 1 1 1

24433767	Bacteria	Pseudomonadota	Betaproteobacteria	Burkholderiales	Oxalobacteraceae	NA	1 0 1 1 1
23777536	Bacteria	Pseudomonadota	Gammaproteobacteria	Xanthomonadales	Xanthomonadaceae	Stenotrophomonas	1 0 1 1 0
21376744	Bacteria	Bacillota	Bacilli	Lactobacillales	NA	NA	1 0 0 0 0
18567411	Bacteria	Pseudomonadota	Gammaproteobacteria	Enterobacterales	Enterobacteriaceae	Leclercia	1 1 1 1 1
18279758	Bacteria	Pseudomonadota	Alphaproteobacteria	Sphingomonadales	Sphingomonadaceae	Sphingomonas	1 1 1 1 1
18096618	Bacteria	Pseudomonadota	Alphaproteobacteria	Rhodobacterales	Paracoccaceae	Paracoccus	1 1 1 1 1
15830629	Bacteria	Actinomycetota	Actinomycetes	Micrococcales	Micrococcaceae	Micrococcus	1 0 0 1 1
15653805	Bacteria	Bacillota	Clostridia	Eubacteriales	Lachnospiraceae	NA	1 0 0 0 0
12376123	Bacteria	Actinomycetota	Actinomycetes	Micrococcales	Micrococcaceae	Kocuria	1 1 1 1 1
12133394	Bacteria	Bacteroidota	Flavobacteriia	Flavobacteriales	Weeksellaceae	NA	1 1 1 1 1
11832944	Bacteria	Pseudomonadota	Alphaproteobacteria	Hyphomicrobiales	Methylobacteriaceae	NA	1 1 1 1 1
11778825	Bacteria	Pseudomonadota	Gammaproteobacteria	Moraxellales	Moraxellaceae	Moraxella	1 1 1 1 1
9917037	Bacteria	Bacteroidota	Cytophagia	Cytophagales	NA	NA	1 1 1 0 1
9917037	Bacteria	Bacteroidota	Cytophagia	NA	NA	NA	1 1 1 1 1
9430242	Bacteria	Pseudomonadota	Alphaproteobacteria	Caulobacterales	Caulobacteraceae	NA	1 0 0 0 0
8834074	Bacteria	Pseudomonadota	Alphaproteobacteria	Hyphomicrobiales	Methylobacteriaceae	Methylobacterium	1 1 1 1 1
8507543	Bacteria	Bacteroidota	Cytophagia	Cytophagales	Hymenobacteraceae	NA	1 1 1 1 1
8023338	Bacteria	Bacteroidota	Cytophagia	Cytophagales	Hymenobacteraceae	Hymenobacter	1 1 1 1 1
7005042	Bacteria	Actinomycetota	Actinomycetes	Micrococcales	Intrasporangiaceae	NA	1 1 1 1 1
5268123	Bacteria	Bacteroidota	Flavobacteriia	Flavobacteriales	Weeksellaceae	Chryseobacterium	1 1 1 1 1
4739621	Bacteria	Pseudomonadota	Betaproteobacteria	Burkholderiales	Burkholderiaceae	Cupriavidus	1 1 1 1 1
4433102	Bacteria	Actinomycetota	Actinomycetes	Micrococcales	Intrasporangiaceae	Janibacter	1 1 1 1 1
4407862	Bacteria	Bacteroidota	Bacteroidia	NA	NA	NA	1 1 1 1 1
4354102	Bacteria	Bacteroidota	Bacteroidia	Bacteroidales	NA	NA	1 1 0 1 1
4249631	Bacteria	Actinomycetota	Actinomycetes	Geodermatophilales	Geodermatophilaceae	Modestobacter	1 1 0 0 1
4196877	Bacteria	Pseudomonadota	Alphaproteobacteria	Rhodospirillales	Acetobacteraceae	Roseomonas	1 0 0 0 0
3683335	Bacteria	Actinomycetota	Actinomycetes	Micrococcales	Micrococcaceae	Rothia	1 1 1 1 1
3651044	Bacteria	Pseudomonadota	Betaproteobacteria	Burkholderiales	Comamonadaceae	Variovorax	1 0 1 0 0
3364490	Bacteria	Actinomycetota	Actinomycetes	Micrococcales	Ornithinimicrobiaceae	NA	1 0 0 0 1
2927152	Bacteria	Pseudomonadota	Gammaproteobacteria	Moraxellales	Moraxellaceae	Psychrobacter	1 1 1 1 1
2858266	Bacteria	Actinomycetota	Actinomycetes	Propionibacteriales	Propionibacteriaceae	Microlunatus	1 1 1 1 1
2789044	Bacteria	Pseudomonadota	Gammaproteobacteria	Enterobacterales	Erwiniaceae	Mixta	1 1 1 1 1
2697058	Bacteria	Pseudomonadota	Gammaproteobacteria	Alteromonadales	NA	NA	1 1 1 1 1
2581926	Bacteria	Actinomycetota	Actinomycetes	Micrococcales	Dermacoccaceae	Dermacoccus	1 1 1 1 1
2408915	Bacteria	Pseudomonadota	Alphaproteobacteria	Hyphomicrobiales	Phyllobacteriaceae	Mesorhizobium	1 0 0 0 0
2119548	Bacteria	Actinomycetota	Actinomycetes	Micrococcales	Ornithinimicrobiaceae	Ornithinimicrobium	1 1 0 0 1

2108076	Bacteria	Pseudomonadota	Alphaproteobacteria	Hyphomicrobiales	Methylobacteriaceae	Methylorubrum	1 1 1 1 1
2106717	Bacteria	Pseudomonadota	Betaproteobacteria	Burkholderiales	Oxalobacteraceae	Pseudoduganella	1 1 1 0 0
2099886	Bacteria	Actinomycetota	Actinomycetes	Micrococcales	Brevibacteriaceae	NA	1 1 1 1 0
2099886	Bacteria	Actinomycetota	Actinomycetes	Micrococcales	Brevibacteriaceae	Brevibacterium	1 1 1 1 0
2044244	Bacteria	Pseudomonadota	Gammaproteobacteria	Enterobacterales	Enterobacteriaceae	Escherichia	1 0 0 0 0
2036773	Bacteria	Pseudomonadota	Betaproteobacteria	Burkholderiales	Comamonadaceae	Comamonas	1 1 0 0 0
1826196	Bacteria	Bacillota	Tissierellia	NA	NA	NA	1 0 0 0 0
1795720	Bacteria	Pseudomonadota	Gammaproteobacteria	Alteromonadales	Shewanellaceae	NA	1 1 1 1 1
1785818	Bacteria	Pseudomonadota	Gammaproteobacteria	Alteromonadales	Shewanellaceae	Shewanella	1 1 1 1 1
1742405	Bacteria	Pseudomonadota	Gammaproteobacteria	Enterobacterales	Enterobacteriaceae	Salmonella	1 1 0 1 1
1673835	Bacteria	Bacillota	Bacilli	Lactobacillales	Lactobacillaceae	Leuconostoc	1 0 0 0 1
1662215	Bacteria	Pseudomonadota	Alphaproteobacteria	Caulobacterales	Caulobacteraceae	Caulobacter	1 1 0 0 0
1590662	Bacteria	Bacillota	Tissierellia	Tissierellales	NA	NA	1 0 0 0 0
1568927	Bacteria	Fusobacteriota	Fusobacteriia	Fusobacteriales	NA	NA	1 1 1 1 1
1568927	Bacteria	Fusobacteriota	NA	NA	NA	NA	1 1 1 1 1
1568927	Bacteria	Fusobacteriota	Fusobacteriia	NA	NA	NA	1 1 1 1 1
1566693	Bacteria	Bacillota	Negativicutes	Veillonellales	NA	NA	1 1 0 0 0
1566693	Bacteria	Bacillota	Negativicutes	Veillonellales	Veillonellaceae	NA	1 1 0 0 0
1448437	Bacteria	Pseudomonadota	Gammaproteobacteria	Xanthomonadales	Xanthomonadaceae	Pseudoxanthomonas	1 1 0 0 1
1365640	Bacteria	Bacillota	Bacilli	Lactobacillales	Aerococcaceae	Aerococcus	1 0 1 1 0
1335044	Bacteria	Actinomycetota	Rubrobacteria	Rubrobacterales	NA	NA	1 0 1 0 0
1335044	Bacteria	Actinomycetota	Rubrobacteria	NA	NA	NA	1 0 1 0 1
1281711	Bacteria	Bacteroidota	Flavobacteriia	Flavobacteriales	Weeksellaceae	Epilithonimonas	1 1 1 1 1
1164329	Bacteria	Bacillota	Bacilli	Bacillales		Exiguobacterium	1 0 1 0 0
1142651	Bacteria	Actinomycetota	Actinomycetes	Micrococcales	Microbacteriaceae	Rathayibacter	1 1 0 1 1
1056327	Bacteria	Bacillota	Bacilli	Lactobacillales	Enterococcaceae	NA	1 1 0 1 0
1015128	Bacteria	Pseudomonadota	Betaproteobacteria	Burkholderiales	Burkholderiaceae	Ralstonia	1 0 1 1 1
923404	Bacteria	Fusobacteriota	Fusobacteriia	Fusobacteriales	Fusobacteriaceae	NA	1 0 0 0 0
918726	Bacteria	Bacillota	Bacilli	Lactobacillales	Enterococcaceae	Enterococcus	1 1 0 1 1
897256	Bacteria	Fusobacteriota	Fusobacteriia	Fusobacteriales	Fusobacteriaceae	Fusobacterium	1 0 0 0 0
850434	Bacteria	Bacteroidota	Chitinophagia	Chitinophagales	NA	NA	1 0 0 0 0
850434	Bacteria	Bacteroidota	Chitinophagia	Chitinophagales	Chitinophagaceae	NA	1 0 0 0 0
850434	Bacteria	Bacteroidota	Chitinophagia	NA	NA	NA	1 0 0 0 0
789318	Bacteria	Bacillota	Clostridia	Eubacteriales	Peptostreptococcaceae	NA	1 0 0 0 0
649206	Bacteria	Pseudomonadota	Alphaproteobacteria	Hyphomicrobiales	Nitrobacteraceae	Rhodopseudomonas	1 0 0 0 0
614260	Bacteria	Pseudomonadota	Alphaproteobacteria	Hyphomicrobiales	Methylobacteriaceae	Microvirga	1 1 0 0 1

611115	Bacteria	Bacillota	Bacilli	Lactobacillales	Lactobacillaceae	Limosilactobacillus	1 0 0 0 0
606165	Bacteria	Pseudomonadota	Alphaproteobacteria	Hyphomicrobiales	Rhizobiaceae	Shinella	1 0 0 0 0
568724	Bacteria	Bacteroidota	Flavobacteriia	Flavobacteriales	Flavobacteriaceae	Capnocytophaga	1 0 0 0 0
561983	Bacteria	Actinomycetota	Actinomycetes	Micrococcales	Microbacteriaceae	Clavibacter	1 1 0 0 1
410778	Bacteria	Campylobacterota	Epsilonproteobacteria	Campylobacterales	Arcobacteraceae	NA	1 1 1 1 1
409880	Bacteria	Bacillota	Bacilli	Bacillales	Bacillaceae	Peribacillus	1 1 1 1 0
391908	Bacteria	Pseudomonadota	Gammaproteobacteria	Enterobacterales	Yersiniaceae	Rahnella	1 1 1 1 1
385880	Bacteria	Bacillota	Bacilli	Lactobacillales	Lactobacillaceae	Latilactobacillus	1 1 1 1 1
344059	Bacteria	Bacteroidota	Bacteroidia	Bacteroidales	Porphyromonadaceae	NA	1 1 0 1 1
344059	Bacteria	Bacteroidota	Bacteroidia	Bacteroidales	Porphyromonadaceae	Porphyromonas	1 1 0 1 1
325807	Bacteria	Pseudomonadota	Alphaproteobacteria	Sphingomonadales	Erythrobacteraceae	Porphyrobacter	1 0 0 0 0
246520	Bacteria	Pseudomonadota	Gammaproteobacteria	Enterobacterales	Morganellaceae	Proteus	1 1 1 1 1
229052	Bacteria	Bacteroidota	Cytophagia	Cytophagales	Spirosomaceae	Dyadobacter	1 0 0 0 0
216431	Bacteria	Pseudomonadota	Betaproteobacteria	Burkholderiales	Oxalobacteraceae	Collimonas	1 0 1 0 0
173556	Bacteria	Bacillota	Bacilli	Bacillales	Staphylococcaceae	Mammaliicoccus	1 1 1 1 1
119209	Bacteria	Pseudomonadota	Gammaproteobacteria	Enterobacterales	Yersiniaceae	Rouxiella	1 0 0 0 0
90232	Archaea	Euryarchaeota	Halobacteria	Halobacteriales	Halobacteriaceae	Halobacterium	1 1 0 0 0
23597355	Bacteria	Pseudomonadota	Gammaproteobacteria	Enterobacterales	Enterobacteriaceae	Enterobacter	0 1 0 0 0
21805113	Bacteria	Pseudomonadota	Alphaproteobacteria	Rhodospirillales	NA	NA	0 1 0 0 0
11296980	Bacteria	Bacillota	Bacilli	Lactobacillales	Streptococcaceae	NA	0 1 0 0 0
9165060	Bacteria	Bacillota	Bacilli	Lactobacillales	Streptococcaceae	Streptococcus	0 1 0 0 0
5895356	Bacteria	Pseudomonadota	Alphaproteobacteria	Rhodospirillales	Acetobacteraceae	NA	0 1 0 0 0
4915138	Bacteria	Actinomycetota	Actinomycetes	Micrococcales	Micrococcaceae	Arthrobacter	0 1 1 0 1
4121904	Bacteria	Bacteroidota	Flavobacteriia	Flavobacteriales	Weeksellaceae	Empedobacter	0 1 0 0 1
3564189	Bacteria	Actinomycetota	Actinomycetes	Mycobacteriales	Gordoniaceae	NA	0 1 1 0 0
3510629	Bacteria	Actinomycetota	Actinomycetes	Mycobacteriales	Gordoniaceae	Gordonia	0 1 1 0 0
2926351	Bacteria	Bacteroidota	Sphingobacteriia	Sphingobacteriales	Sphingobacteriaceae	Pedobacter	0 1 0 0 1
2697733	Bacteria	Pseudomonadota	Gammaproteobacteria	Enterobacterales	Enterobacteriaceae	Kosakonia	0 1 0 0 0
2218173	Bacteria	Bacteroidota	Bacteroidia	Bacteroidales	Prevotellaceae	NA	0 1 0 0 1
2022135	Bacteria	Bacteroidota	Bacteroidia	Bacteroidales	Prevotellaceae	Prevotella	0 1 0 0 1
1697606	Bacteria	Bacillota	Bacilli	Lactobacillales	Lactobacillaceae	Lactobacillus	0 1 0 1 0
1436449	Bacteria	Bacillota	Tissierellia	Tissierellales	Peptoniphilaceae	NA	0 1 0 0 0
642730	Bacteria	Fusobacteriota	Fusobacteriia	Fusobacteriales	Leptotrichiaceae	NA	0 1 0 0 1
631631	Bacteria	Pseudomonadota	Gammaproteobacteria	Enterobacterales	Morganellaceae	NA	0 1 0 1 1
565778	Bacteria	Pseudomonadota	Gammaproteobacteria	Enterobacterales	Enterobacteriaceae	Kluyvera	0 1 0 0 0
432979	Bacteria	Pseudomonadota	Betaproteobacteria	Burkholderiales		Rhizobacter	0 1 0 0 0

396124	Bacteria	Bacteroidota	Flavobacteriia	Flavobacteriales	Weeksellaceae	Cloacibacterium	0 1 1 1 1
291712378	Bacteria	Pseudomonadota	Gammaproteobacteria	Pseudomonadales	NA	NA	0 0 1 0 0
291506545	Bacteria	Pseudomonadota	Gammaproteobacteria	Pseudomonadales	Pseudomonadaceae	NA	0 0 1 0 0
47278720	Bacteria	Actinomycetota	Actinomycetes	Mycobacteriales	NA	NA	0 0 1 0 0
19715001	Bacteria	Pseudomonadota	Betaproteobacteria	Burkholderiales	Oxalobacteraceae	Massilia	0 0 1 1 0
16075272	Bacteria	Bacteroidota	Flavobacteriia	NA	NA	NA	0 0 1 1 0
6114626	Bacteria	Actinomycetota	Actinomycetes	Actinomycetales	Actinomycetaceae	Actinomyces	0 0 1 0 0
2918686	Bacteria	Pseudomonadota	Gammaproteobacteria	Xanthomonadales	Xanthomonadaceae	Xanthomonas	0 0 1 0 0
1645995	Bacteria	Bacillota	Bacilli	Lactobacillales	Aerococcaceae	NA	0 0 1 0 0
1443713	Bacteria	Actinomycetota	Actinomycetes	Propionibacteriales	Propionibacteriaceae	Tessaracoccus	0 0 1 1 0
1070726	Bacteria	Actinomycetota	Actinomycetes	Micrococcales	Microbacteriaceae	Curtobacterium	0 0 1 0 0
911676	Bacteria	Actinomycetota	Actinomycetes	Actinomycetales	Actinomycetaceae	Schaalia	0 0 1 0 0
195987	Bacteria	Deinococcota	Deinococci	Thermales	NA	NA	0 0 1 0 0
195987	Bacteria	Deinococcota	Deinococci	Thermales	Thermaceae	NA	0 0 1 0 0
155510	Bacteria	Pseudomonadota	Gammaproteobacteria	Xanthomonadales	Rhodanobacteraceae	Dyella	0 0 1 0 1
16075272	Bacteria	Bacteroidota	Flavobacteriia	Flavobacteriales	NA	NA	0 0 0 1 0
4908830	Bacteria	Pseudomonadota	Alphaproteobacteria	Sphingomonadales	Sphingomonadaceae	Sphingobium	0 0 0 1 0
2813982	Bacteria	Actinomycetota	Actinomycetes	Micrococcales	Dermabacteraceae	NA	0 0 0 1 0
545867	Bacteria	Actinomycetota	Actinomycetes	Nakamurellales	Nakamurellaceae	Nakamurella	0 0 0 1 0
361682	Bacteria	Actinomycetota	Actinomycetes	Micrococcales	Beutenbergiaceae	NA	0 0 0 1 0
268222	Bacteria	Bacteroidota	Bacteroidia	Bacteroidales	Tannerellaceae	NA	0 0 0 1 0
7768137	Bacteria	Actinomycetota	Actinomycetes	Geodermatophilales	NA	NA	0 0 0 0 1
7768137	Bacteria	Actinomycetota	Actinomycetes	Geodermatophilales	Geodermatophilaceae	NA	0 0 0 0 1
2926982	Bacteria	Actinomycetota	Actinomycetes	Micrococcales	Dermacoccaceae	NA	0 0 0 0 1
2150050	Bacteria	Actinomycetota	Actinomycetes	Geodermatophilales	Geodermatophilaceae	Blastococcus	0 0 0 0 1
2096533	Bacteria	Bacillota	Bacilli	Lactobacillales	Streptococcaceae	Lactococcus	0 0 0 0 1
1167029	Bacteria	Actinomycetota	Actinomycetes	Micrococcales	Ornithinimicrobiaceae	Serinicoccus	0 0 0 0 1
873653	Bacteria	Bacillota	Bacilli	Lactobacillales	Carnobacteriaceae	NA	0 0 0 0 1
301495	Bacteria	Pseudomonadota	Betaproteobacteria	Burkholderiales	Sphaerotilaceae	Caldimonas	0 0 0 0 1
185754	Bacteria	Bacteroidota	Cytophagia	Cytophagales	Hymenobacteraceae	Pontibacter	0 0 0 0 1
166985	Bacteria	Bacillota	Clostridia	Eubacteriales	Eubacteriales Family XIII. Incertae Sedis	NA	0 0 0 0 1

1.3 Classification models with functional profiles

1.3.1 Grid Search Parameters

**Parameter**	**Values**
Decision Tree and Extra Trees Classifier	
Criterion	entropy, gini index
Number of estimators	10, 50, 100, 300, 500, 750, 1200
Max depth	3, 5, 8, 10, 12, 15, 20, 30, 35, 40
Support Vector Classifier	
Kernel	linear, poly, rbf
Degree	2, 3, 4, 5, 6, 7, 8
K-nearest neighbors Classifier	
Number of neighbors	2, 3, 4, 5, 6, 7, 8, 9, 10, 11, 12, 13, 14, 15
Weights	uniform, distance
Multi-Layer Perceptron Classifier	
Hidden layer sizes	(100,), (200,), (50, 50), (20, 20, 20)
Activation function	relu, tanh
Batch size	auto, 50, 100
Solver	adam, sgd
Maximum iterations	1000, 3000, 5000
Early stopping	False, True
Learning rate	constant, adaptive

Table S3. Parameter values for different classifiers

1.3.2 Grid Search Top Results

Model	Accuracy	Balanced Accuracy	F1 Score
PROKKA_MLP	0.764	0.726	0.73
PROKKA_VC(soft)	0.727	0.654	0.651
Mifaser4_SVC	0.745	0.663	0.63
Mifaser4_MLP	0.745	0.642	0.621
UNIPROT_VC(hard)	0.691	0.643	0.617
UNIPROT_RandomForest	0.691	0.636	0.601
KEGG_MLP	0.691	0.627	0.595
PROKKA_VC(hard)	0.655	0.605	0.588
UNIPROT_MLP	0.691	0.606	0.58
Mifaser3_VC(soft)	0.691	0.614	0.578
Mifaser3_SVC	0.655	0.606	0.578
UNIPROT_ExtraTrees	0.655	0.596	0.57
Mifaser3_VC(hard)	0.655	0.612	0.563
KEGG_ExtraTrees	0.691	0.592	0.562
KEGG_VC(soft)	0.618	0.556	0.559

Table S4. Top 15 performing models based on F1 Score without 5-fold cross-validation. Refer to the full document to see all results.

1.3.3 Performance by city

We evaluated the performance of the best models for each annotation tool stratified by city. For the evaluation, we used 5-fold cross-validation and evaluated using F1 macro score.

City	Mifaser4	KEGG	Prokka
AKL	0.774	0.767	0.774
BER	0.529	0.489	0.539
BOG	0.928	0.811	0.805
DEN	0.742	0.823	0.865
DOH	0.593	0.509	0.594
ILR	0.753	0.773	0.776
LIS	0.708	0.667	0.701
NYC	0.802	0.749	0.684
SAC	0.631	0.682	0.623
TOK	0.733	0.768	0.749
BAL	0.491	0.491	0.571
MIN	0.496	0.496	0.496
SAN	0.488	0.539	0.641
SAO	0.811	0.701	0.726
VIE	0.488	0.489	0.489
ZRH	0.489	0.485	0.486
Average	0.654	0.64	0.657

Table S5. Performance by city using F1 score

2 MOST ABUNDANT GENERA AND PHYLA

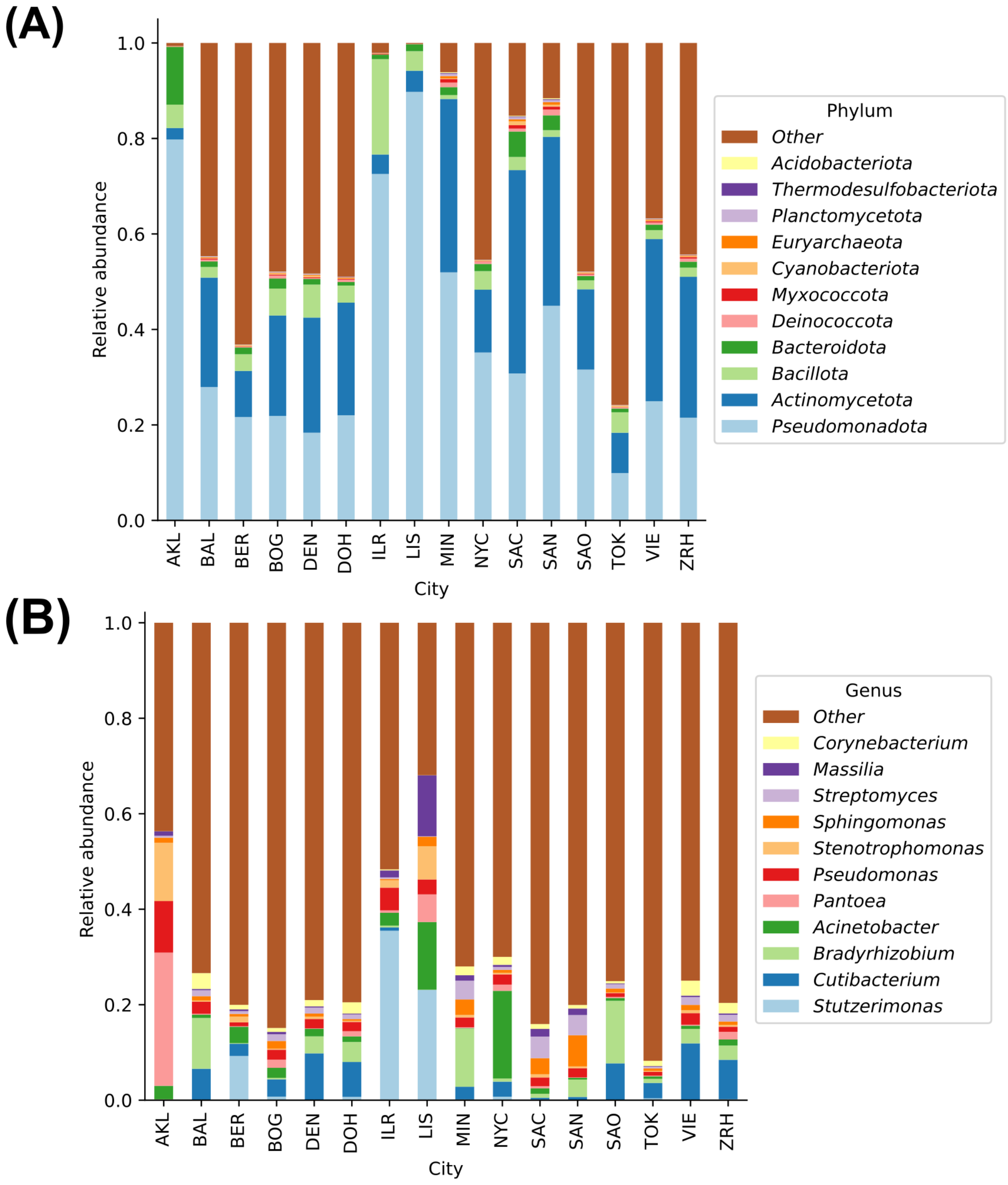

Figure S1: Relative read abundance of the most abundant microbial taxa per city at the phylum and species level, using NCBI Taxonomy as reference. **(A)** The most dominant phyla are within the Bacteria domain, with the exception of Euryarchaeota, which are archaeal organisms. The four most abundant phyla identified are consistent with the results of the urban metagenomic samples from Danko et al. (2021). **(A)** The most representative genera belong to the phylum Pseudomonadota, with the exception of Cutibacterium, Streptomyces and Corynebacterium, which belong to Actinomycetota. More than 50% of the genera found in Auckland (AKL) and Lisbon (LIS) belong to one of the eleven genera represented in the plot, reflecting the low alpha diversity found in these cities as shown in Figure 2a in the main article.

3 MULTI-VARIABLE LOGISTIC REGRESSION MODEL

We aim to set up a classification model starting from a set of data consisting in 365 items, where for each item we have 20,448 attributes. Each item should be classified in one of the following 16 classes: AKL, BAL, BER, BOG, DEN, DOH, ILR, LIS, MIN, NYC, SAC, SAN, SAO, TOK, VIE, ZRH.

The classification model consists in selecting J of the 20,448 attributes, performing a principal component analysis on the natural logarithm of the J selected attributes, and taking the first N principal components as input for the multi-variable logistic regression classification algorithm.

We applied this classification scheme using several values for J and N and observed that classification accuracy can reach up to 88 percent when using, for example $J = 5,000$ attributes and $N = 70$ principal components. Additionally, we found that the accuracy achieved by this classification model does not improve by taking larger values for J.

A more detailed description of the classification scheme under consideration is provided below.

1. Take the first J attributes from the total of 20,448 attributes of each of the 365 items. More precisely, consider the matrix X of dimensions $365 \times J$. Each row of X represents one of the 365 items to be classified with the help of the first J attributes for each item. However, the attributes considered in X are the natural logarithm of the original data. This is to say that the elements of X are of the form $\log(d_{ij} + 1)$ were d_{ij} is an entry in the original data set. It is important to note that we did not standardize the entries in matrix X.
2. Compute the matrix C which is the correlation matrix of X. We call U the matrix whose columns are the eigenvalues of C ordered according to the decreasing order of the corresponding eigenvalues. Each column of U correspond to a principal component of the transformed data matrix X. The quotient of the sum of the first N eigenvalues and the sum of the total J eigenvalues is the percentage of the variation of the data explained by the first N principal components. For a given value of J we choose N in such a way that with the first N principal components we obtain the maximum accuracy (see figure S5). With this N, we explain about 91% of the variation of the data.
3. The next step is to compute the matrix $Z = X \times U$ which gives the coordinates relative to the principal components of the 365 items from which we are setting up the classification model (see figure S2).
4. Each row of Z has a label serving as an identifier to the class of which the corresponding item belongs to.
5. From matrix Z we take a random sample Z_{tr} of size 274 to be fed to a calcification algorithm. The complement of Z_{tr} relative to Z we call Z_{tst} and will serve to test the accuracy of the classification algorithm. As classification algorithm we used the multi-variable logistic regression algorithm.
6. Once a classification model has been produced in step 5, we tested its accuracy by computing the percentage of correct classifications for each of the $91 = 365 - 274$ items in matrix Z_{tst} (see figure S4).
7. Steps 5 and 6 were repeated 500 times. Each time these two steps were performed we obtained an index of the accuracy classification model. The statistical behavior of these 500 indices inform us as to what can we expect of the classification model here considered when applying it in practice.

We let J take the values 2,500, 5,000, 7,500, 10,000 and 12,500. For each of these values for J we performed steps 5 and 6 a total number of 500 times. The distribution of the performance index are reported in table S6 and in figure S3. We notice that the best results are obtained when $J = 10,000$ since in this

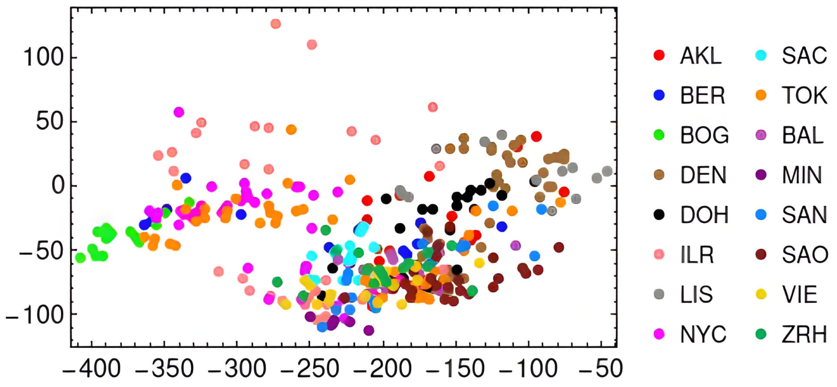

Figure S2: Here we see the distribution of the data when plotted against the first two principal components when taken the first $J = 5,000$ data attributes.

case we have a mean performance of 90% of correct classifications of the elements in the random sample of size 91 taken from the population of 365 items. A comparison of the performance of this classification model for the above five values of J suggest that the performance does not improve as J takes larger values. Moreover, we did not go beyond the value of 12,500 for J due to the limited computation capability of a typical personal computer. For each value of J we ran the classification model for distinct values of N and selected the value of N with the highest accuracy. Finally, this classification scheme was put to work in the Mathematica software system and we found that while the multi-variable logistic regression algorithm performed satisfactorily, other algorithms for classification did not give as good results.

J	N	%	accuracy
2,500	80	92	0.88
5,000	70	91	0.88
7,500	80	92	0.88
10,000	110	91	0.90
12,500	50	79	0.88

Table S6. In this table we report the accuracy achieved by the classification model here considered for distinct values of J an N. In the third column we report the percentage of the variation of the data explained by the first N principal components.

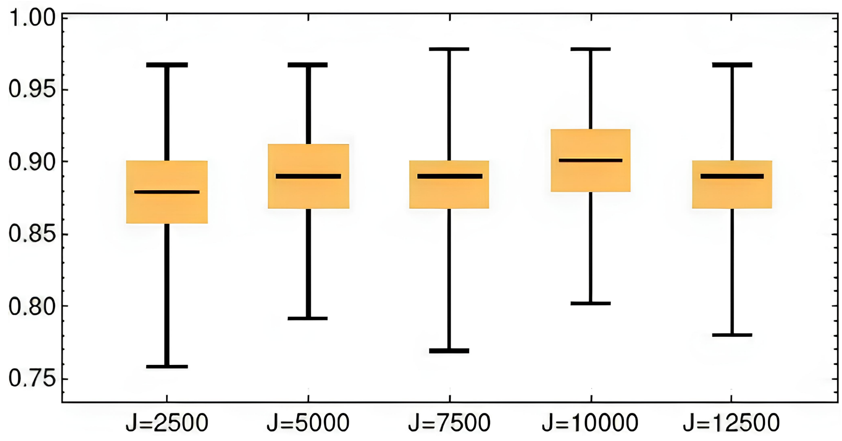

Figure S3: Box and whisker plot of 500 runs for distinct values of J and for the best value of N.

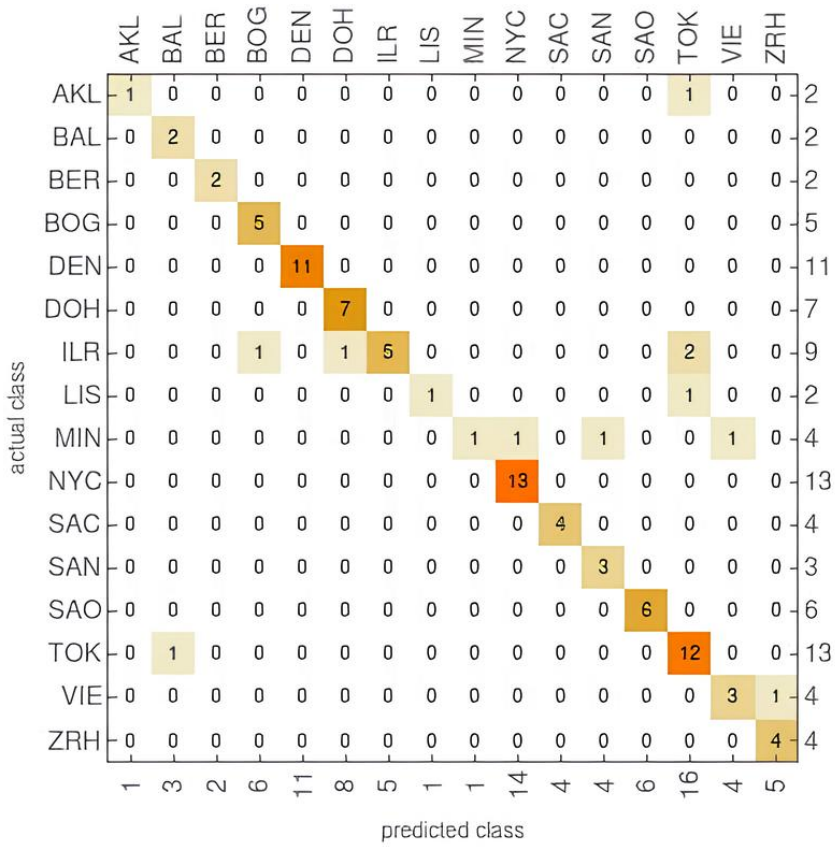

Figure S4: Confusion matrix for an accuracy of 0.879, when $J = 5,000$.

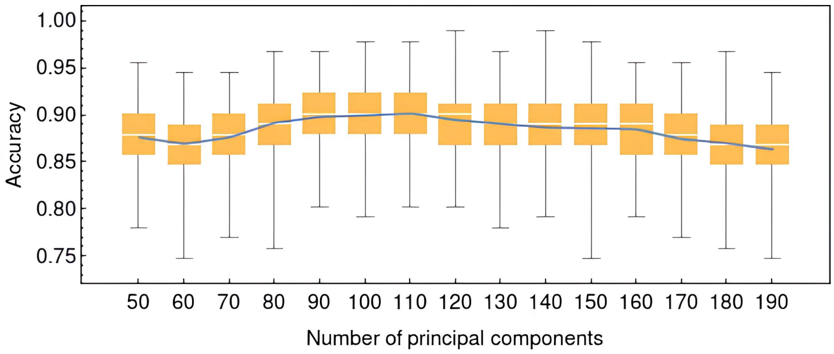

Figure S5: For $J = 10,000$ we have the performance of the classification model for distinct values of N. A maximum accuracy occurs when $N = 110$.
